# Supplementary material for: Genomic Surveillance of Listeria monocytogenes in Taiwan, 2014 to 2019
Source: Microbiol Spectr. 2022 Oct 12;10(6):e01825-22. doi: 10.1128/spectrum.01825-22 (PMC9769603; doi:10.1128/spectrum.01825-22)
Supplement: Supplemental file 1 — Tables S1 and S3 and Fig. S1. Download spectrum.01825-22-s0001.pdf, PDF file, 0.3 MB [file spectrum.01825-22-s0001.pdf]

SUPPLEMENTAL MATERIAL

Genomic surveillance of *Listeria monocytogenes* in Taiwan, 2014-2019

Yu-Huan Tsai[1,*], Alexandra Moura[2,3,*], Zi-Qi Gu[1], Jui-Hsien Chang[4], Ying-Shu Liao[4], Ru-Hsiou Teng[4], Kuo-Yao Tseng[1], Dai-Ling Chang[1], Wei-Ren Liu[1], Yu-Tsung Huang[5,6], Alexandre Leclercq[2,3], Hsiu-Jung Lo[7,8,9], Marc Lecuit[2,3,10,#], Chien-Shun Chiou[4, #]

[1] Laboratory of Host-Microbe Interactions and Cell Dynamics, Institute of Microbiology and Immunology, National Yang Ming Chiao Tung University, Taipei, Taiwan

[2] Institut Pasteur, Université Paris Cité, Inserm U1117, Biology of Infection Unit, Paris, France

[3] Institut Pasteur, National Reference Centre and WHO Collaborating Centre *Listeria*, Paris, France

[4] Center for Diagnostics and Vaccine Development, Centers for Disease Control, Taichung, Taiwan

[5] Departments of Laboratory Medicine and Internal Medicine, National Taiwan University Hospital, National Taiwan University College of Medicine, Taipei, Taiwan

[6] Graduate Institute of Clinical Laboratory Sciences and Medical Biotechnology, National Taiwan University, Taipei, Taiwan

[7] National Institute of Infectious Disease and Vaccinology, National Health Research Institutes, Miaoli, Taiwan

[8] School of Dentistry, China Medical University, Taichung, Taiwan

[9] Department of Biological Science and Technology, National Yang Ming Chiao Tung University, Hsinchu, Taiwan

[10] Necker-Enfants Malades University Hospital, Division of Infectious Diseases and Tropical Medicine, Institut Imagine, APHP, Paris, France

Table S1. Number of *Listeria monocytogenes*-positive food samples collected per region and food type in this study.

Product	Taipei	New Tapei	Taichung	Hsinchu	Total
Raw ground pork	18/27 (67%)	19/28 (68%)	32/62 (52%)	-	69/117 (59%)
Raw chicken	5/23 (22%)	8/27 (30%)	12/63 (19%)	-	25/113 (22%)
Mixed salads	-	-	-	0/50 (0%)	0/50 (0%)
Total	23/50 (46%)	27/55 (49%)	44/125 (35%)	0/50 (0%)	94/290 (32%)

Table S2. Characteristics of *Listeria monocytogenes* isolates retrieved in this study (N=493).

[extended excel file]

Table S3. Previously reported cgMLST types detected in this study (*n*=10).

cgMLST type (clonal complex, serogroup)	source(s) (this study)	source(s) (other studies)	countries (other studies)	source lab	Min. cgMLST similarity (%)
Lineage 1					
L1-SL1-ST1-CT2384 (CC1, IVb)	H	H,F,PE	US	FDA,CDC	99.6%
L1-SL224-ST845-CT21 (CC224, IIb)	H		MX,US	FDA,SENESICA	99.9%
L1-SL87-ST87-CT58 (CC87, IIb)	H,F	H,F,PE	AU,CA,CN,CO,DE,FR,SK,US	CDC,CFIA,DI,FDA,IP,RKI	99.6%
Lineage 2					
L2-SL101-ST101-CT4370 (CC101, IIa)	H	n.a.	n.a.	n.a.	99.7%
L2-SL155-ST155-CT4364 (CC155, IIa)	H,F	n.a.	n.a.	n.a.	99.6%
L2-SL16-ST16-CT5516 (CC8, IIa)	F	F	CA	CFIA	99.7%
L2-SL177-ST177-CT5041 (CC177, IIa)	H	H	KH	CDC-TW	99.8%
L2-SL378-ST378-CT4349 (CC19, IIa)	H,F	H	KH	CDC-TW	99.6%
L2-SL8-ST8-CT5167 (CC8, IIa)	H	n.a.	n.a.	n.a.	99.9%
L2-SL9-ST9-CT5537 (CC9,IIa, IIc)	F	H,F	FR	IP	99.8%

Source abbreviations: H, human; F, food; PE, food production environment.

Country abbreviations: AU, Australia, CA, Canada; CN, China; CO, Colombia; FR, France; KH, Cambodia; MX, Mexico; SK, South Korea; US, United States of America; n.a., not available.

Source lab abbreviations: CDC, Centers for Disease Control, United States; CDC-TW, Centers for Disease Control Taiwan; CFIA, Canadian Food Inspection Agency, Canada; DI, Doherty Institute, Australia; FDA, Food and Drug Administration, United States; IP, Institut Pasteur, France; RKI, Robert Koch Institut, Germany; SENESICA, Servicio Nacional de Sanidad Inocuidad y Calidad Agroalimentaria, Mexico; n.a., not available.

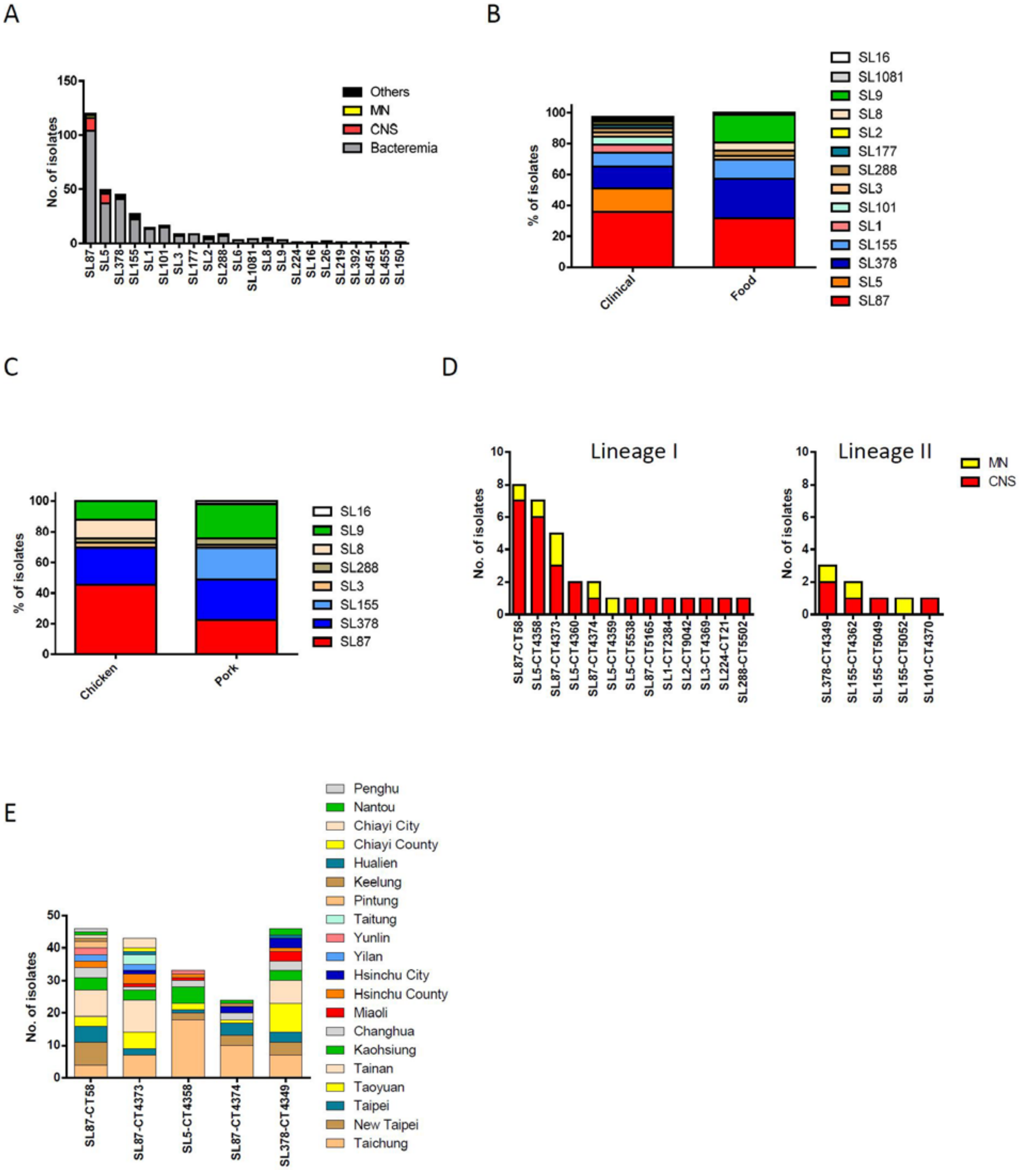

Figure S1. Distribution of *L. monocytogenes* major cgMLST sublineages (SLs) and types (CTs) in clinical and food sources in Taiwan. (A) No. isolates per SL and clinical form (2018-2019). (B) Percentage of larger SLs in clinical and food sources (2014-2019). (C) Percentage of larger SLs per food type (2014-2019). (D) Prevalence of cgMLST types in maternal-neonatal and CNS infections, for which the clinical information was available (n=327 isolates). (E) Distribution of major CTs of clinical isolates in different administrative regions of Taiwan. Abbreviations: CNS, central nervous system infection; MN, maternal-neonatal infections.
